# Optimal α/β Ratio for Biologically Effective Dose-Based Prediction of Radiation-Induced Peritumoral Brain Edema in Meningioma

**DOI:** 10.3390/cancers18030448

**Published:** 2026-01-30

**Authors:** Shin-Woong Ko, Yu Deok Won, Byeong Jin Ha, Jin Hwan Cheong, Je Il Ryu, Seung Woo Hong, Kyueng-Whan Min, Myung-Hoon Han

**Affiliations:** 1 Department of Neurosurgery, Hanyang University Guri Hospital, 153 Gyeongchun-ro, Guri 11923, Republic of Korea; pakky100@naver.com (S.-W.K.); hidma823@hanmail.net (Y.D.W.); bbddock@hanmail.net (B.J.H.); cjh2324@hanyang.ac.kr (J.H.C.); ryujeil@hanyang.ac.kr (J.I.R.); 2 Department of Neurosurgery, Hanyang University Medical Center, 222-1 Wangsimni-ro, Seongdong-gu, Seoul 04763, Republic of Korea; enokaiser@gmail.com; 3 Department of Pathology, Uijeongbu Eulji Medical Center, School of Medicine, Eulji University, Uijeongbu 11759, Republic of Korea

**Keywords:** meningioma, peritumoral brain edema, biologically effective dose, α/β ratio, radiotherapy, stereotactic radiosurgery

## Abstract

**Background**: Peritumoral brain edema (PTBE) is the most frequent complication for intracranial meningiomas following radiotherapy, yet no clinically validated biologically effective dose (BED) threshold capable of reliably predicting PTBE has currently been established. Although conventional radiobiological models typically assume an α/β ratio of 2–4 for benign meningiomas, whether these values accurately reflect the dose–response characteristics underlying radiation-induced PTBE remains unclear. **Methods**: We analyzed 67 intact meningiomas in the convexity, parasagittal, or falcine regions treated with primary linear accelerator (LINAC)-based radiotherapy. The BED values were recalculated using α/β ratios ranging from 2 to 20, and receiver operating characteristic (ROC) analyses were performed to identify the optimal BED thresholds for predicting PTBE. The most informative α/β ratio was defined as the value yielding the highest Youden’s J statistic. **Results**: The ROC analyses showed that an assumed α/β ratio of 14 provided the highest discriminative accuracy for predicting PTBE in the overall cohort and markedly superior performance in patients younger than 70 years (area under the curve (AUC) 0.945; Youden’s J = 0.871). The optimal BED threshold for predicting PTBE was approximately 41 Gy (α/β = 14), corresponding to ~18 Gy in a single fraction and ~5.8 Gy per fraction in a five-fraction regimen. **Conclusions**: The BED values calculated using α/β ratios near 14 provide the most reliable estimate of PTBE risk following primary LINAC-based radiotherapy for convexity, parasagittal, and falcine meningiomas. Maintaining prescription doses below this threshold may help reduce the likelihood of PTBE in this patient population.

## Simple Summary

Peritumoral brain edema (PTBE) remains the most frequent complication following radiotherapy for intracranial meningiomas, yet a clinically reliable biologically effective dose (BED) threshold to predict this toxicity has not been defined. This study systematically evaluated a range of assumed α/β ratios to identify the most appropriate radiobiological model for predicting PTBE in convexity, parasagittal, and falcine meningiomas treated with primary LINAC-based radiotherapy. The analysis demonstrated that an α/β ratio of approximately 14 provides the highest predictive accuracy, corresponding to an optimal BED threshold of around 41 Gy. These results indicate that maintaining prescribed doses below this level may reduce the risk of radiation-induced PTBE, particularly in patients younger than 70 years. The findings propose a refined BED-based framework that could improve individualized dose planning and the radiobiological understanding of PTBE formation in meningioma radiotherapy.

## 1. Introduction

Meningiomas are the most common extra-axial intracranial tumors and constitute a significant proportion of benign brain neoplasms. Microsurgical resection remains the standard treatment for symptomatic lesions; however, complete removal is not always feasible because of factors such as tumor size, location, or involvement of critical neurovascular structures, as well as patient-related factors. Therefore, radiation therapy is widely used as a primary option for small (<3 cm) asymptomatic tumors or those situated at the skull base. Although radiotherapy is generally regarded as safe, symptomatic peritumoral brain edema (PTBE) represents the most frequently associated complication, occurring in approximately 6–35% of treated intracranial meningiomas [1]. When PTBE develops after radiotherapy, it can lead to new neurological symptoms and prolonged corticosteroid use. In more severe presentations, patients may require inpatient management, while rare cases of life-threatening deterioration may even lead to death [1,2].

Multiple clinical and dosimetric factors have been proposed as predictors of post-radiotherapy PTBE for meningioma, including larger tumor volume, higher prescription doses, parasagittal or falcine location, and variations in the tumor–brain interface [3,4]. Among these, the radiation dose has been most consistently recognized as a key determinant of PTBE risk, with higher marginal or biologically effective doses associated with an increased likelihood of edema formation [1,3]. Despite accumulating clinical experience with stereotactic and fractionated radiotherapy for intracranial meningiomas, a clinically validated biologically effective dose (BED) threshold that reliably predicts the development of PTBE has not yet been defined. Although radiobiological modeling commonly assumes an α/β ratio of approximately 2–4 for benign meningiomas, whether BED values derived from these conventional parameters accurately represent the vascular, inflammatory, and microenvironmental responses that underlie PTBE formation remains unclear. Thus, previous studies have yet to conclusively establish the BED level that most strongly correlates with radiation-induced PTBE, or the α/β ratio that best characterizes this risk [1,3,5,6,7].

Therefore, to address these uncertainties, this study aimed to evaluate whether BED values could serve as reliable predictors of PTBE following radiotherapy for intracranial meningiomas. Given the elevated risk of peritumoral edema after radiation treatment in convexity, parasagittal, and parafalcine meningiomas, we restricted our analysis to lesions arising in these locations [3,7,8]. Moreover, focusing on a uniform set of tumor sites minimized location-related confounding factors and allowed a more consistent assessment of those associated with radiation-induced edema. Furthermore, to reduce heterogeneity arising from postoperative tissue alterations, pre-existing edema, or changes in the tumor–brain interface, we included only intact meningiomas treated with primary linear accelerator (LINAC)-based radiotherapy. By concentrating on this anatomically and clinically homogeneous subset, we minimized the confounding factors and enabled a more rigorous assessment of the relationship between assumed α/β ratios, corresponding BED values, and subsequent PTBE development. Building on this rationale, this study aimed to systematically compare BED metrics calculated across a broad range of α/β ratios and to examine the associated predictive performance for radiation-induced PTBE in convexity, parasagittal, and falcine meningiomas undergoing primary LINAC-based radiotherapy.

## 2. Materials and Methods

### 2.1. Patient Selection

This study included patients enrolled in the institutional NOVALIS Registry, a previously described prospectively maintained database that systematically captures clinical, radiological, and dosimetric information for all individuals treated with LINAC-based radiotherapy at our center [4,9]. Prospective design ensures standardized data collection and consistent longitudinal follow-up, enabling reliable evaluation of treatment-related outcomes such as PTBE. Moreover, by incorporating all consecutive patients treated during the study period, the registry minimizes selection bias and strengthens the internal validity of the cohort. A total of 177 patients with 187 intracranial meningiomas underwent LINAC-based radiotherapy at our institution between July 2014 and August 2024.

**Figure 1:**
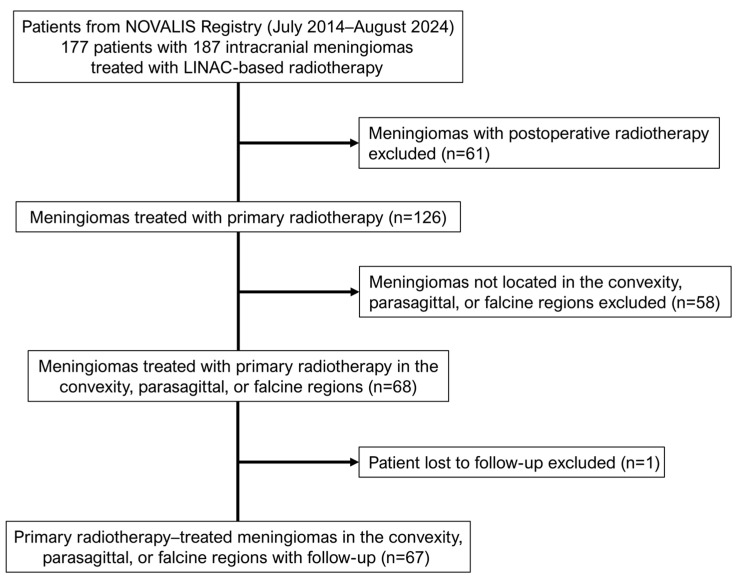
Flow diagram of the patient selection process for the study cohort. Abbreviation: LINAC, linear accelerator.

To reduce heterogeneity and identify factors influencing the development of radiation-induced PTBE within a more comparable clinical context, we applied the following exclusion criteria: (1) meningiomas treated with postoperative radiotherapy, as prior surgical manipulation could introduce confounding factors related to tissue healing, alteration of the tumor–brain interface, and pre-existing edema (*n* = 61); (2) meningiomas not located in the convexity, parasagittal, or falcine regions, as these sites have the highest propensity for PTBE and share relatively uniform anatomical characteristics and PTBE risk profiles (*n* = 58); (3) patients lacking sufficient clinical or imaging follow-up (*n* = 1). After applying these criteria, 65 patients with 67 intact meningiomas treated with primary LINAC-based radiotherapy in the convexity, parasagittal, or falcine regions were included in the final analytical cohort (Figure 1). All study patients had a minimum clinical follow-up of 6 months and at least one post-treatment follow-up imaging study (computed tomography (CT) or magnetic resonance imaging (MRI)).

### 2.2. Imaging and Clinical Assessment

All meningiomas and cases of PTBE were confirmed by two experienced radiologists through independent assessments on brain MRI. Post-radiotherapy PTBE was defined as either a newly developed edema or a measurable increase in the volume or extent of a pre-existing edema on the follow-up MRI after radiotherapy, typically appearing as hyperintensity on T2-weighted or FLAIR sequences [10]. All patients who developed PTBE exhibited corresponding neurological symptoms, which ranged from mild deficits to marked clinical deterioration.

### 2.3. Radiation Technique and Treatment Planning

As previously reported, all radiotherapy procedures were performed using the NOVALIS Tx platform (Varian Medical Systems, Palo Alto, CA, USA; Brainlab, Feldkirchen, Germany) [4,9]. Detailed descriptions of the radiotherapy procedures, including immobilization, image guidance, and treatment planning, are available in our previous reports [4,9]. The gross tumor volume (GTV) was defined as the contrast-enhancing tumor on the T1-weighted MRI. Since the cohort consisted solely of patients without prior surgical resection, all lesions were presumed to be grade I meningiomas according to the World Health Organization (WHO) classification, and the clinical target volume (CTV) was set equal to the GTV. The planning target volume (PTV) was generated by applying a symmetric 0–2 mm expansion to the CTV, according to institutional standards.

Radiotherapy fractionation regimens followed stereotactic protocols: hypofractionated stereotactic radiotherapy (hf-SRT) was delivered in 6–10 fractions; hypofractionated stereotactic radiosurgery (hf-SRS) in 2–5 fractions; single-fraction stereotactic radiosurgery (SRS) as a one-session treatment. To enable comparison across heterogeneous fractionation schemes, the BED was calculated for each patient using the linear–quadratic model (BED = nd × [1 + d/3]), with an initial α/β ratio of 3 for meningioma tissue.

### 2.4. Statistical Methods

Age was analyzed as a dichotomized variable using a cutoff of 70 years (<70 vs. ≥70), a threshold commonly adopted in previous brain tumor radiotherapy studies to define an elderly subgroup and to reflect age-related differences in treatment tolerance [11,12,13].

Hazard ratios (HRs) and associated 95% confidence intervals (CIs) were estimated using both univariate and multivariate Cox proportional hazards models to evaluate factors related to the development of PTBE after primary LINAC-based radiotherapy for convexity, parasagittal, and falcine meningiomas. The cumulative incidence of PTBE over time was assessed using Kaplan–Meier methods, with patient stratification based on relevant predictive variables.

To determine which assumed α/β ratio yields the most informative BED parameter for predicting radiation-induced PTBE, we recalculated BED values across a range of α/β ratios (2 to 20) and constructed receiver operating characteristic (ROC) curves for each ratio. For each ROC curve, the optimal threshold was identified as the point with the minimal Euclidean distance to the upper-left corner, computed as $\sqrt{{\left(1-sensitivity\right)}^{2}+{\left(1-specificity\right)}^{2}}$. From these curves, we obtained the area under the curve (AUC) and associated diagnostic metrics, including sensitivity, specificity, and Youden’s J statistic (sensitivity + specificity − 1). To identify the most informative BED parameter among the range of α/β ratios, we selected the BED value corresponding to the α/β ratio that yielded the highest Youden’s J statistic [14].

A value of *p* < 0.05 was considered statistically significant. All statistical analyses were performed using R software version 4.3.3 and SPSS for Windows, version 24.0 (IBM, Chicago, IL, USA).

## 3. Results

### 3.1. Characteristics of the Study Patients

A total of 65 patients with 67 intracranial meningiomas located in the convexity, parasagittal, or falcine regions who received primary LINAC-based radiotherapy were included in the analysis (Figure 1).

PTBE occurred in 16 cases (23.9%). The mean age was 68.1 years, and 76.1% of the patients were female. The median imaging follow-up duration was 678 days, with a mean follow-up of 854.9 days. The mean PTV was significantly larger in the PTBE (+) group (21.1 cc) than in the PTBE (−) group (6.3 cc). Most patients were treated with hf-SRS; 13.4% received single-fraction SRS, and 9.0% underwent hf-SRT. The mean marginal dose was 27.2 Gy, and the mean BED (α/β = 3) was 88.2 Gy. Detailed baseline characteristics are summarized in Table 1.

### 3.2. Predictors of PTBE After Radiotherapy

In the univariate Cox regression analysis, older age and a larger PTV were significantly associated with PTBE development (Table 2).

However, in the multivariate analysis, BED (α/β = 3) emerged as the only significant independent predictor of PTBE (HR, 1.06; 95% CI, 1.01–1.12; *p* = 0.017), despite not being significant in the univariate model. Notably, neither age nor PTV remained statistically significant after adjustment for covariates.

Given this discrepancy between univariate and multivariate results, potential multicollinearity among BED, PTV, and fractionation was evaluated using variance inflation factors derived from a corresponding multivariable linear regression model in which BED (α/β = 3) was specified as the dependent variable. The results of this collinearity assessment are summarized in Supplementary Table S1. Mild collinearity was observed for PTV (VIF = 2.03) and fractionation (VIF = 2.10), indicating a modest degree of shared variance but no severe multicollinearity that would compromise the stability of the multivariate Cox regression model.

### 3.3. Determination of Optimal BED Cutoffs for Predicting PTBE

To clarify which assumed α/β ratio yields the most reliable BED estimation for predicting radiation-induced PTBE, we systematically compared ROC-based performance metrics across α/β values ranging from 2 to 20. Table 3 presents the corresponding optimal BED thresholds and diagnostic indices.

Across all patients, both the AUC and Youden’s J values increased with higher α/β ratios, reaching a plateau within the 10–16 range. Within this interval, the AUC values reached 0.716–0.733, and Youden’s J ranged from 0.475 to 0.495, consistently outperforming values obtained at lower or higher α/β ratios. Notably, α/β ratios of 14 and 15 yielded the highest Youden’s J (0.495), with a sensitivity of 0.750 and a specificity of 0.745. The dose-per-fraction values corresponding to commonly applied fractionation schemes (1, 3, 5, 8, 10, 15, and 20 fractions) for the optimal BED cutoffs (α/β ratios: 2–20) used to predict radiation-induced PTBE can be found in Supplementary Table S2.

Meanwhile, the discriminative performance varied substantially with the assumed α/β ratio among patients younger than 70 years. This subgroup demonstrated markedly stronger predictive performance than the overall cohort, exhibiting consistently higher AUC values across all α/β ratios. The α/β range of 11–15 showed the strongest discriminative capability, with AUC values of 0.932–0.945 and Youden’s J values of 0.839–0.871. The α/β ratios of 12–14 yielded the highest Youden’s J (0.871), corresponding to a sensitivity of 1.000 and a specificity of 0.871 (Table 3).

In contrast, no meaningful discriminative performance was observed in patients aged ≥70 years. The AUC values remained low (0.468–0.600), and Youden’s J indices were modest (0.145–0.327) across all α/β ratios. Additionally, none of the BED thresholds in this age group reached statistical significance (Table 3).

### 3.4. Optimal BED Threshold with an α/β Ratio of 14 for Predicting Radiation-Induced PTBE

**Figure 2:**
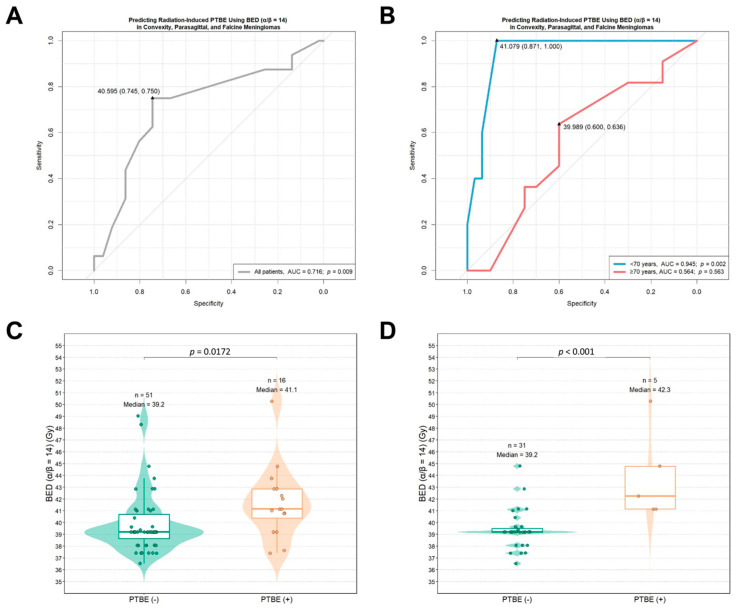
Receiver-operating characteristic (ROC) analyses and distribution of the biologically effective doses (BEDs) (α/β = 14) associated with radiation-induced peritumoral brain edema (PTBE) in convexity, parasagittal, and falcine meningiomas treated with primary linear accelerator (LINAC)-based radiotherapy. (**A**) ROC curve evaluating the predictive performance of the BED (α/β = 14) for PTBE in the overall cohort; (**B**) ROC curves stratified by patient age (<70 years vs. ≥70 years); (**C**) distribution of the BEDs (α/β = 14) in the overall cohort, comparing patients with and without PTBE; (**D**) distribution of BED values (α/β = 14) among patients younger than 70 years, according to PTBE status. Abbreviations: AUC, area under the curve; BED, biologically effective dose; LINAC, linear accelerator; PTBE, peritumoral brain edema.

Based on these findings, we selected the optimal BED value derived from an assumed α/β ratio of 14, which yielded the highest Youden’s J value in both the overall cohort and the subgroup of patients younger than 70 years, as the most informative threshold for predicting LINAC-based radiation-induced PTBE in convexity, parasagittal, and falcine meningiomas. In the overall cohort, the optimal BED cutoff of 40.595 Gy (α/β = 14) produced a sensitivity of 0.745 and a specificity of 0.750 (Figure 2A).

**Figure 3:**
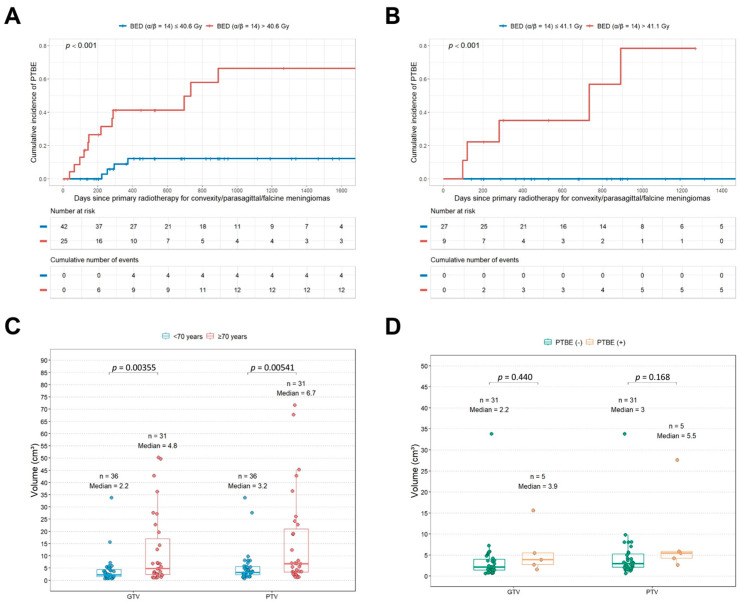
Cumulative incidence of PTBE according to the BED thresholds and volumetric parameters in convexity, parasagittal, and falcine meningiomas treated with primary LINAC-based radiotherapy. (**A**) Kaplan–Meier estimates of PTBE incidence stratified by a BED threshold of 40.6 Gy (α/β = 14) in the overall cohort; (**B**) Kaplan–Meier curves of the PTBE incidence among patients younger than 70 years, stratified by a BED threshold of 41.1 Gy (α/β = 14); (**C**) comparison of the gross tumor volumes (GTVs) and planning target volumes (PTVs) between patients aged <70 years and those aged ≥70 years; (**D**) distribution of the GTVs and PTVs according to PTBE occurrence within the <70-year subgroup. Abbreviations: BED, biologically effective dose; GTV, gross tumor volume; LINAC, linear accelerator; PTBE, peritumoral brain edema; PTV, planning target volume.

In patients younger than 70 years, the optimal BED threshold of 41.079 Gy, calculated using an α/β ratio of 14, demonstrated markedly superior predictive accuracy for radiation-induced PTBE, yielding an AUC of 0.945 (Figure 2B). We observed that BED (α/β ratio = 14) values were significantly higher in patients who developed PTBE than in those without PTBE, in both the overall cohort and the subgroup of patients younger than 70 years (Figure 2C,D). Meanwhile, the BED values (α/β = 14) across all patients were significantly higher in the PTBE development group than in both the local control and tumor progression groups (analysis of variance (ANOVA), *p* = 0.026; Figure S1). When stratified by the optimal BED cutoffs, patients with BEDs above the thresholds exhibited a significantly higher cumulative hazard of PTBE in both the overall cohort and the younger subgroup (Figure 3A,B).

Larger GTVs or PTVs are known to predispose patients to developing PTBE, independent of radiation dose effects. Therefore, we examined whether baseline tumor size influenced the predictive reliability of the BED thresholds. Indeed, younger patients tended to have smaller tumor volumes and considerably less inter-patient variability than older patients (Figure 3C). Moreover, neither the GTV nor the PTV differed significantly between the PTBE (–) and PTBE (+) groups within the <70-year subgroup (Figure 3D).

### 3.5. PTV and Age Thresholds for Predicting Radiation-Induced PTBE

We additionally evaluated the predictive value of the PTV. The optimal PTV cutoff for the overall cohort was 11.110 cm^3^, corresponding to an AUC of 0.806 (Figure S2A). When stratified by this threshold, patients with a PTV > 11.1 cm^3^ had a markedly higher cumulative incidence of PTBE than those with a PTV ≤ 11.1 cm^3^ (Figure S2B). We also assessed the predictive value of age and identified 72.5 years as the optimal threshold (Figure S2C). When patients were stratified by age (cutoff at 72 years), those older than 72 years demonstrated a significantly higher cumulative hazard of PTBE following primary radiotherapy for convexity, parasagittal, and falcine meningiomas (Figure S2D).

### 3.6. Subgroup Analysis in Patients with Pre-Existing PTBE

A subgroup analysis was performed in patients with pre-existing peritumoral brain edema prior to radiotherapy (*n* = 8), all of whom were neurologically asymptomatic at baseline (Supplementary Table S3). Post-radiotherapy PTBE developed in four patients (50.0%). Although no statistically significant differences were observed between patients with and without post-radiotherapy PTBE, those who developed PTBE tended to be older and to have larger tumor volumes.

## 4. Discussion

We systematically evaluated a broad range of assumed α/β ratios (2–20) to identify the ratio that yielded the most reliable BED estimate for predicting radiation-induced PTBE in patients with convexity, parasagittal, and falcine meningiomas treated with primary LINAC-based radiotherapy. Our analysis demonstrated that α/β ratios near 14 produced the most stable and discriminative predictive performance, with robust accuracy across the overall cohort and exceptionally strong predictive accuracy among patients younger than 70 years. Specifically, a BED value of approximately 41 Gy (α/β = 14) was identified as the optimal threshold for predicting primary radiation-induced PTBE occurrence. This threshold corresponds to an estimated physical dose of nearly 18 Gy in a single fraction or approximately 5.8 Gy per fraction in a five-fraction schedule. Although higher radiation doses are typically recommended for more aggressive tumors, such as meningiomas classified as grades II–III using the WHO guidelines, the fact that single-fraction doses approaching 18 Gy are widely used in clinical practice for these higher-grade lesions lends support to the credibility of our observation that an upper limit of approximately 18 Gy in a single-fraction regimen may be reasonable [15]. These results imply that, for patients younger than 70 years with convexity, parasagittal, or falcine meningiomas, maintaining prescription doses below this threshold may help mitigate the risk of PTBE, whereas exceeding this threshold may increase the risk. In contrast, using the BED presented minimal predictive utility for patients aged 70 years or older. The volumes of meningiomas in patients younger than 70 years were generally smaller and less variable than those in older patients (Figure 3C), reducing size-related confounding and allowing the dose–response relationship to emerge more clearly. In contrast, greater volume heterogeneity in older patients, potentially related to comorbidities or limited surgical candidacy, may reduce the discriminative value of dose-based predictors such as BED. In addition, the GTV and PTV distributions were relatively uniform between the PTBE (–) and PTBE (+) groups within the <70-year group (Figure 3D). This finding further supports the interpretation that the BED threshold provides a more dose-specific and reliable predictor of PTBE in this age group. Accordingly, we propose that the BED cutoff calculated using an assumed α/β ratio near 14 is the most reliable discriminator for predicting primary LINAC-based radiation-induced PTBE in convexity, parasagittal, and falcine meningiomas, particularly in patients younger than 70 years.

An accurate specification of the α/β ratio is essential when calculating the BED to predict radiation-induced PTBE in meningiomas, as the chosen α/β value directly determines the dose per fraction for any given fractionation scheme. For example, when delivering a biologically equivalent dose corresponding to 17 Gy in a single fraction, the required dose per fraction in a five-fraction regimen varies substantially according to the assumed α/β ratio: 6.89 Gy for α/β = 3, 5.80 Gy for α/β = 10, and 5.43 Gy for α/β = 14. Thus, if an α/β ratio of 10 is assumed, clinicians should select a five-fraction regimen of 5.8 Gy per fraction. However, if the optimal α/β ratio for predicting PTBE is actually closer to 14, as suggested by our findings, the same 5.8 Gy fraction size already approximates or slightly exceeds the BED threshold associated with increased PTBE risk when calculated with α/β = 14. Thus, mis-specifying the α/β ratio may inadvertently lead to the administration of a dose per fraction within a high-risk range for PTBE, even when the regimen is intended to be conservative. These observations underscore the importance of determining an appropriate α/β ratio when planning fractionated radiotherapy for meningiomas.

The observation that meningiomas exhibit an α/β value around 3, whereas the prediction of radiation-induced PTBE was best achieved using an α/β value near 14, can be explained within a radiobiological framework. Meningiomas are slow-proliferating tumors, and the response characteristics of these tumors are consistent with low fractionation sensitivity, for which a relatively small α/β value is generally considered appropriate. Indeed, previous studies have estimated α/β values for benign meningiomas in the range of approximately 2–4 [5,16]. However, PTBE reflects a normal tissue response rather than a tumor cell response, involving radiation-induced vascular injury, blood–brain barrier (BBB) disruption, increased microvascular permeability, immune activation, and white matter demyelination [17,18,19]. These mechanisms correspond to early-responding tissues, characterized by high α/β ratios, consistent with evidence that BBB breakdown, neuroinflammation, demyelination, and vasogenic edema can occur within weeks to months after cranial irradiation [20,21,22,23]. Since early-responding tissues typically exhibit high α/β ratios, the observed α/β ≈ 14 in this study is biologically plausible for predicting PTBE occurrence after radiation [24,25]. Moreover, PTBE does not merely reflect residual swelling following tumor cell death; rather, PTBE reflects radiation-induced responses in the surrounding normal brain tissue, vasculature, and immune microenvironment. Although the structurally slow-cycling meningioma exhibits a low α/β ratio, the edema-forming reactions arising from the adjacent normal brain, vascular endothelium, and the local microenvironment may exhibit substantially higher α/β characteristics [20,21,23]. This distinction underscores that the α/β ratio governing tumor control may differ fundamentally from that governing normal tissue injury, highlighting the need to model the tumor response and toxicity as separate radiobiological processes. Indeed, whereas dose-escalation strategies for tumor control may reasonably assume a low α/β ratio to guide fractionation decisions, modeling edema formation or other forms of normal tissue injury may require different α/β values to more accurately reflect tissue-specific radiosensitivity. Thus, applying distinct α/β assumptions in this context enhances the precision of toxicity-risk prediction and facilitates more individualized treatment planning. In summary, effective treatment planning for meningiomas should not assume equivalence between the α/β ratio of the tumor and that of the surrounding normal tissues. The findings in this study provide empirical support for the notion that these radiobiological parameters may indeed differ.

Larger PTVs are intrinsically predisposed to developing PTBE after radiotherapy for meningioma due to several microanatomical and physiological factors. A greater irradiated volume encompasses a larger proportion of peritumoral white matter and vascular structures, increasing the likelihood of BBB disruption, microvascular injury, cytokine-mediated inflammatory responses, injury at the brain–meningioma interface, and subsequent vasogenic edema. These mechanisms are supported by previous studies showing a strong correlation between irradiated volume and the risk or severity of radiation-induced edema in intracranial tumors [7,9,26,27].

In addition to these radiobiological considerations, our findings also suggest that age-related factors may partly account for the limited predictive value of the BED in patients aged 70 years or older. As noted above, meningiomas in the older cohort tended to be substantially larger and more heterogeneous in volume, which likely introduces significant confounding when predicting PTBE based solely on dose metrics. In addition, older patients may be more likely to harbor pre-existing cerebrovascular pathology, including cerebral small vessel disease and chronic microangiopathy related to long-standing hypertension, diabetes, and other vascular risk factors, which may increase BBB vulnerability and susceptibility to edema formation [28]. Consistent with this, clinical studies of radiation-induced brain injury and radionecrosis have identified advanced age and underlying vascular comorbidities as important susceptibility factors, independent of the prescribed dose [29].

Our study has several limitations. First, despite the prospective registry, the analysis remains retrospective, with inherent limitations. Second, the relatively small sample size may limit statistical power, particularly in subgroup analyses; therefore, validation of these data in larger multi-institutional cohorts is warranted. Nevertheless, our restrictive inclusion criteria (no prior surgery and focus on convexity/parasagittal/falcine tumors) likely reduced heterogeneity, minimizing confounding from surgical manipulation or variable tumor–brain interface geometry. Third, pathological confirmation was unavailable because the study only included patients treated with primary radiotherapy. Consequently, the cohort may have contained a mixture of WHO grade I, II, and possibly III meningiomas, and this histological heterogeneity could have influenced the observed incidence of PTBE following radiotherapy. Finally, given the relatively small cohort and the exploration of multiple α/β ratios and BED thresholds, a degree of overfitting cannot be excluded. The lack of internal or external validation further underscores the need for confirmation of these findings in larger, independent cohorts.

## 5. Conclusions

We evaluated the predictive performance of the BED across a wide range of assumed α/β ratios to identify dose parameters most closely associated with the development of PTBE in convexity, parasagittal, and falcine meningiomas treated with primary LINAC-based radiotherapy. Our findings indicate that BED values calculated with α/β ratios around 14 provide the best discriminative performance, with an optimal BED threshold of approximately 41 Gy demonstrating notably high predictive accuracy for PTBE, particularly among patients younger than 70 years. This threshold corresponds to a physical dose of roughly 18 Gy in a single-fraction regimen or about 5.8 Gy per fraction in a five-fraction regimen. Collectively, these findings suggest that maintaining prescribed doses below this BED range may help reduce the risk of clinically significant PTBE in patients receiving primary radiotherapy for convexity, parasagittal, and falcine meningiomas. However, this threshold is intended to support risk stratification rather than to mandate specific dose prescriptions, and prospective validation is required before routine clinical implementation.

## Figures and Tables

**Table 1:** Characteristics of patients treated with primary LINAC-based radiation for convexity, parasagittal, or falcine meningiomas.

Characteristics	PTBE (−)	PTBE (+)	Total	*p*-Value
Number (%)	51 (76.1)	16 (23.9)	67	
Sex, female, *n* (%)	41 (80.4)	10 (62.5)	51 (76.1)	0.143
Age, mean ± SD, y	66.8 ± 10.5	72.2 ± 13.5	68.1 ± 11.4	0.101
Age < 70 years, *n* (%)	31 (60.8)	5 (31.3)	36 (53.7)	0.039
Imaging follow-up period, median (IQR), days	531.0 (269.0–1268.0)	978.0 (144.3–1696.0)	678.0 (204.0–1337.0)	0.507
Imaging follow-up period, mean ± SD, days	821.4 ± 697.6	961.7 ±844.8	854.9 ± 731.1	0.507
BMI, mean ± SD, kg/m^2^	24.2 ± 4.0	24.5 ± 3.0	24.2 ± 3.7	0.764
Location, *n* (%)				0.327
Convexity	28 (54.9)	11 (68.8)	39 (58.2)	
Parasagittal or parafalcine	23 (45.1)	5 (31.3)	28 (41.8)	
GTV, mean ± SD, cc	5.3 ± 9.0	15.6 ± 15.6	7.8 ± 11.7	0.001
PTV, mean ± SD, cc	6.3 ± 10.5	21.1 ± 19.9	9.9 ± 14.6	<0.001
Marginal radiation dose, mean ± SD, Gy	27.2 ± 3.7	27.4 ± 6.1	27.2 ± 4.4	0.826
Fractionation, *n* (%)				0.006
SRS	5 (9.8)	4 (25.0)	9 (13.4)	
hf-SRS (2–5 fx)	44 (86.3)	8 (50.0)	52 (77.6)	
hf-SRT (6–10 fx)	2 (3.9)	4 (25.0)	6 (9.0)	
Dose per fraction, mean ± SD, Gy	6.9 ± 3.8	8.4 ± 5.9	7.3 ± 4.4	0.248
BED (α/β = 3), mean ± SD, Gy	86.4 ± 14.8	94.0 ± 22.9	88.2 ± 17.2	0.126
Tumor progression, *n* (%)	6 (11.8)	0	6 (9.0)	0.150
Past medical history, *n* (%)				
Hypertension	26 (51.0)	9 (56.3)	35 (52.2)	0.713
Diabetes	10 (19.6)	2 (12.5)	12 (17.9)	0.518

BED: biologically effective dose; BMI: body mass index; Fx, fraction; GTV: gross tumor volume; Gy: gray; hf-SRT: hypofractionated stereotactic radiotherapy; hf-SRS: hypofractionated stereotactic radiosurgery; IQR: interquartile range; LINAC: linear accelerator; PTBE: peritumoral brain edema; PTV: planning target volume; SD: standard deviation; SRS: stereotactic radiosurgery.

**Table 2:** Univariate and multivariate Cox regression analyses of predictors for PTBE in patients with intracranial convexity, parasagittal, and falcine meningiomas treated with LINAC-based radiotherapy.

	Univariate Analysis		Multivariate Analysis	
Variable	HR (95% CI)	*p*-Value	HR (95% CI)	*p*-Value
Sex				
Male	Reference		Reference	
Female	0.41 (0.15–1.14)	0.086	0.67 (0.16–2.77)	0.577
Age (per 1 year increase)	1.06 (1.01–1.11)	0.030	1.04 (0.98–1.09)	0.169
BMI (per 1 BMI increase)	1.01 (0.88–1.17)	0.868	1.03 (0.84–1.27)	0.767
PTV (per 1 cc increase)	1.04 (1.02–1.06)	<0.001	1.04 (0.99–1.09)	0.154
BED (α/β = 3) (per 1 Gy increase)	1.02 (1.00–1.04)	0.114	1.06 (1.01–1.12)	0.017
Fractionation (per 1 fraction increase)	1.17 (0.92–1.50)	0.207	1.10 (0.64–1.87)	0.734
Hypertension				
No	Reference		Reference	
Yes	1.13 (0.42–3.03)	0.813	1.92 (0.56–6.57)	0.298
Diabetes				
No	Reference		Reference	
Yes	0.66 (0.15–2.92)	0.588	0.16 (0.02–1.31)	0.087

BED, biologically effective dose; BMI, body mass index; CI, confidence interval; Gy, gray; HR, hazard ratio; PTV, planning target volume.

**Table 3:** ROC-based evaluation of optimal BED cutoffs (α/β ratios: 2–20) for predicting radiation-induced PTBE in all patients and age-stratified subgroups (<70 years and ≥70 years) with convexity, parasagittal, and falcine meningiomas.

α/β	AUC	*p*-Value	Optimal BED (Gy)	Sensitivity	Specificity	Youden’s J	1 fx Dose ≤ (Gy)	5 fx Dose/fx ≤ (Gy)
All patients								
2	0.550	0.551	119.840	0.500	0.804	0.304	14.51	5.99
3	0.556	0.503	89.427	0.500	0.804	0.304	14.95	5.98
4	0.559	0.476	74.550	0.500	0.804	0.304	15.38	5.98
5	0.593	0.267	65.096	0.500	0.784	0.284	15.71	5.95
6	0.602	0.219	59.672	0.500	0.804	0.304	16.16	5.98
7	0.662	0.051	50.457	0.688	0.667	0.355	15.62	5.60
8	0.663	0.050	48.025	0.688	0.667	0.355	16.00	5.64
9	0.675	0.036	46.798	0.688	0.686	0.374	16.51	5.72
10	0.726	0.007	43.785	0.812	0.667	0.479	16.51	5.61
11	0.730	0.006	42.577	0.812	0.667	0.479	16.83	5.63
12	0.733	0.005	41.075	0.812	0.667	0.479	17.00	5.60
13	0.718	0.009	41.217	0.750	0.725	0.475	17.54	5.72
14	0.716	0.009	40.595	0.750	0.745	0.495	17.85	5.75
15	0.722	0.008	39.565	0.750	0.745	0.495	17.99	5.73
16	0.719	0.009	38.136	0.750	0.725	0.475	17.96	5.64
17	0.680	0.031	38.487	0.625	0.804	0.429	18.45	5.75
18	0.677	0.034	37.910	0.625	0.804	0.429	18.63	5.75
19	0.682	0.029	37.394	0.625	0.804	0.429	18.80	5.74
20	0.654	0.065	36.749	0.625	0.804	0.429	18.90	5.72
Patients younger than 70 years								
α/β	AUC	*p*-value	Optimal BED (Gy)	Sensitivity	Specificity	Youden’s J	1 fx dose ≤ (Gy)	5 fx dose/fx ≤ (Gy)
2	0.745	0.082	144.118	0.800	0.871	0.671	16.01	6.66
3	0.745	0.082	105.562	0.800	0.871	0.671	16.36	6.60
4	0.758	0.067	95.031	0.800	0.903	0.703	17.60	6.95
5	0.790	0.040	80.775	0.800	0.903	0.703	17.75	6.83
6	0.790	0.040	70.271	0.800	0.903	0.703	17.75	6.66
7	0.906	0.004	50.457	1.000	0.710	0.710	15.62	5.60
8	0.906	0.004	48.025	1.000	0.710	0.710	16.00	5.64
9	0.919	0.003	46.798	1.000	0.742	0.742	16.51	5.72
10	0.919	0.003	45.160	1.000	0.742	0.742	16.83	5.74
11	0.939	0.002	44.440	1.000	0.839	0.839	17.28	5.81
12	0.939	0.002	43.327	1.000	0.871	0.871	17.58	5.83
13	0.932	0.002	42.415	1.000	0.871	0.871	17.86	5.85
14	0.945	0.002	41.079	1.000	0.871	0.871	17.98	5.81
15	0.932	0.002	39.565	1.000	0.839	0.839	17.99	5.73
16	0.919	0.003	38.136	1.000	0.806	0.806	17.96	5.64
17	0.719	0.120	39.565	0.600	0.935	0.535	18.79	5.88
18	0.706	0.143	38.700	0.600	0.935	0.535	18.89	5.84
19	0.706	0.143	37.926	0.600	0.935	0.535	18.98	5.81
20	0.623	0.385	36.749	0.600	0.903	0.503	18.90	5.72
Patients older than 70 years								
α/β	AUC	*p*-value	Optimal BED (Gy)	Sensitivity	Specificity	Youden’s J	1 fx dose ≤ (Gy)	5 fx dose/fx ≤ (Gy)
2	0.468	0.773	98.075	0.273	0.950	0.223	13.04	5.34
3	0.473	0.804	72.450	0.182	1.000	0.182	13.32	5.26
4	0.473	0.804	61.838	0.182	1.000	0.182	13.85	5.31
5	0.477	0.836	55.470	0.182	1.000	0.182	14.34	5.36
6	0.486	0.901	54.792	0.545	0.600	0.145	15.38	5.65
7	0.536	0.741	51.450	0.545	0.600	0.145	15.80	5.68
8	0.536	0.741	50.169	0.455	0.700	0.155	16.43	5.81
9	0.550	0.650	44.939	0.909	0.300	0.209	16.11	5.56
10	0.600	0.364	43.785	0.727	0.600	0.327	16.51	5.61
11	0.586	0.433	42.577	0.727	0.600	0.327	16.83	5.63
12	0.595	0.386	41.075	0.727	0.600	0.327	17.00	5.60
13	0.577	0.483	40.719	0.636	0.600	0.236	17.41	5.67
14	0.564	0.563	39.989	0.636	0.600	0.236	17.67	5.69
15	0.582	0.457	40.237	0.545	0.750	0.295	18.19	5.80
16	0.582	0.457	39.659	0.545	0.750	0.295	18.43	5.82
17	0.591	0.409	39.150	0.545	0.750	0.295	18.66	5.83
18	0.591	0.409	38.617	0.545	0.750	0.295	18.86	5.83
19	0.591	0.409	38.084	0.545	0.750	0.295	19.03	5.83
20	0.595	0.386	37.605	0.545	0.750	0.295	19.19	5.82

AUC, area under the curve; BED, biologically effective dose; Fx, fraction; Gy, gray; PTBE, peritumoral brain edema; ROC, receiver operating characteristic curve; Youden’s J, Youden index (J = sensitivity + specificity − 1).

## Data Availability

The datasets analyzed during the current study are not publicly available due to patient privacy and ethical restrictions but are available from the corresponding author upon reasonable request.

## Supplementary Materials

The following supporting information can be downloaded at: https://www.mdpi.com/article/10.3390/cancers18030448/s1, Figure S1. Violin and box plots of the distribution of biologically effective dose (BED; α/β = 14) across clinical outcome groups in patients with convexity, parasagittal, and falcine meningiomas treated with primary LINAC-based radiotherapy. Figure S2. ROC analyses and cumulative incidence curves of the predictive performance of PTV and age thresholds for radiation-induced PTBE in convexity, parasagittal, and falcine meningiomas treated with primary LINAC-based radiotherapy. Table S1. Multivariable linear regression analysis for assessment of collinearity among covariates using BED (α/β = 3) as the dependent variable. Table S2. ROC-based evaluation of optimal BED cutoffs (α/β ratios: 2–20) for predicting radiation-induced PTBE, with corresponding dose-per-fraction values for commonly applied fractionation schemes (1, 3, 5, 8, 10, 15, and 20 fractions) in all patients and age-stratified subgroups with convexity, parasagittal, and falcine meningiomas. Table S3. Comparison between patients with and without the development of PTBE after radiotherapy among those with pre-existing edema prior to radiotherapy.

## Abbreviations

BED	Biologically effective dose
PTBE	Peritumoral brain edema
LINAC	Linear accelerator
SRS	Stereotactic radiosurgery
hf-SRS	Hypofractionated stereotactic radiosurgery
hf-SRT	Hypofractionated stereotactic radiotherapy
GTV	Gross tumor volume
CTV	Clinical target volume
PTV	Planning target volume
ROC	Receiver operating characteristic
AUC	Area under the curve
HR	Hazard ratio
CI	Confidence interval
WHO	World Health Organization
BBB	Blood–brain barrier
MRI	Magnetic resonance imaging
CT	Computed tomography
ANOVA	Analysis of variance
