# Supplementary material for: Optimal α/β Ratio for Biologically Effective Dose-Based Prediction of Radiation-Induced Peritumoral Brain Edema in Meningioma
Source: Cancers (Basel). 2026 Jan 30;18(3):448. doi: 10.3390/cancers18030448 (PMC12896445; doi:10.3390/cancers18030448)
Supplement: Supplementary file 1 [file cancers-18-00448-s001.zip › cancers-4083496-supplementary.pdf]

Supplementary Materials

Optimal α/β Ratio for Biologically Effective Dose-Based Prediction of Radiation-Induced Peritumoral Brain Edema in Meningioma

Shin-Woong Ko, Byeong Jin Ha, Yu Deok Won, Jin Hwan Cheong, Je Il Ryu, Seung Woo Hong, Kyueng-Whan Min, Myung-Hoon Han

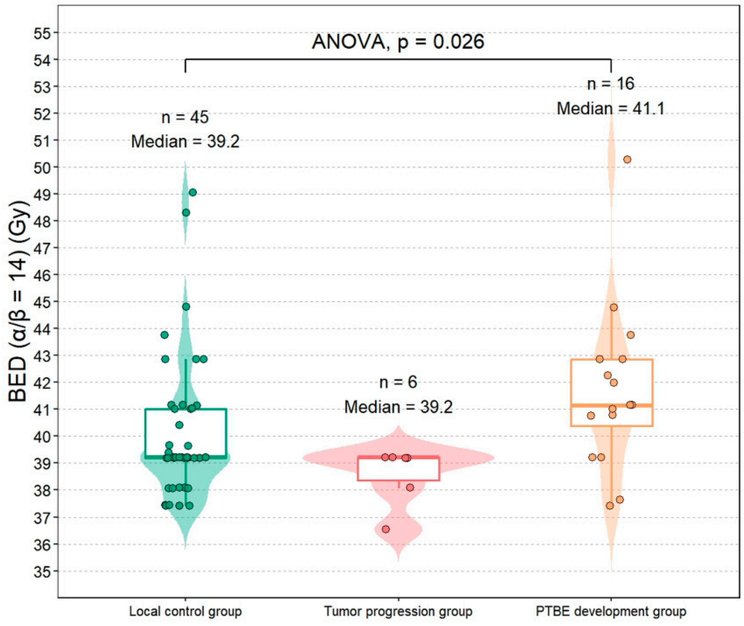

Supplementary Figure S1. Violin and box plots of the distribution of biologically effective dose (BED; α/β = 14) across clinical outcome groups in patients with convexity, parasagittal, and falcine meningiomas treated with primary LINAC-based radiotherapy. BED (α/β = 14) values in three clinically distinct subgroups: the local control group (green), the tumor progression group (pink), and the PTBE development group (orange). Violin plots represent kernel density estimates of BED distributions, overlaid with boxplots with the median values and interquartile ranges. Individual patient values are displayed as jittered points to illustrate within-group variability. Group-level sample sizes (n) and median BED values are annotated above each violin. A one-way analysis of variance (ANOVA) demonstrated a statistically significant difference in BED values among the three groups.

Abbreviation: RBANS, Repeatable Battery for the Assessment of Neuropsychological Status.

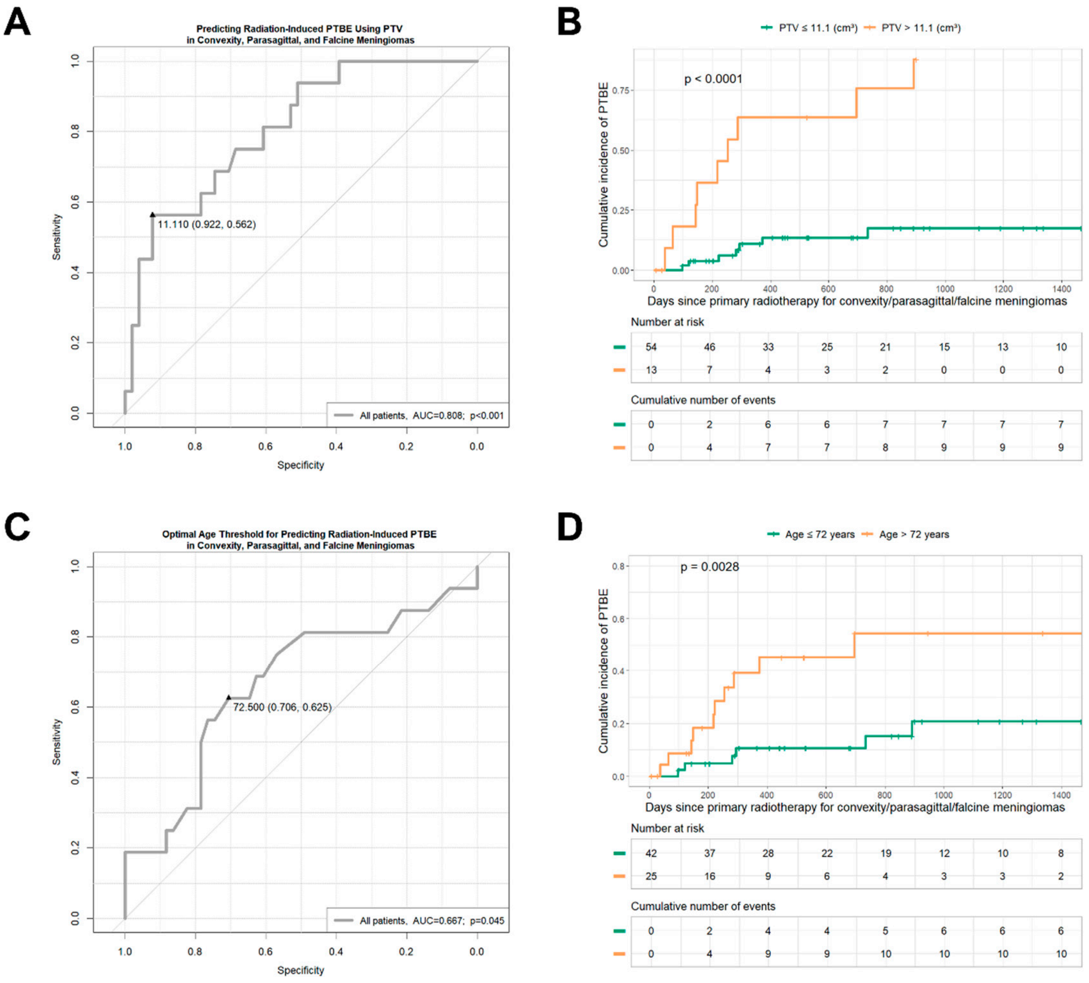

Supplementary Figure S2. ROC analyses and cumulative incidence curves of the predictive performance of PTV and age thresholds for radiation-induced PTBE in convexity, parasagittal, and falcine meningiomas treated with primary LINAC-based radiotherapy.

(A) ROC curve for the optimal PTV cutoff value for predicting radiation-induced PTBE; (B) Kaplan–Meier estimates for the cumulative incidence of PTBE stratified by the optimal PTV cutoff (≤11.1 cm³ vs. >11.1 cm³); (C) ROC curve for the optimal age cutoff value for predicting radiation-induced PTBE; (D) Kaplan–Meier curves comparing the cumulative

incidence of PTBE between patients aged ≤72 years and those older than 72 years.

Abbreviations: AUC, area under the curve; LINAC, linear accelerator; PTBE, peritumoral brain edema; PTV, planning target volume; ROC, receiver operating characteristic.

Supplementary Table S1. Multivariable linear regression analysis for assessment of collinearity among covariates using BED ($\alpha/\beta = 3$) as the dependent variable.

	Multivariable linear regression analysis		
Variable	β (95% CI)	p-value	VIF
Sex			
Male	Reference		
Female	2.80 (-4.27–9.86)	0.430	1.162
Age (per 1 year increase)	0.06 (-0.22–0.35)	0.655	1.385
BMI (per 1 BMI increase)	-0.09 (-0.89–0.71)	0.824	1.151
PTV (per 1 cc increase)	0.54 (0.28–0.80)	<0.001	2.027
Fractionation (per 1 fraction increase)	-8.45 (-10.25–6.66)	<0.001	2.100
Hypertension			
No	Reference		
Yes	-0.17 (-6.46–6.13)	0.958	1.303
Diabetes			
No	Reference		
Yes	-0.91 (-10.24–8.42)	0.846	1.572

BED, biologically effective dose; BMI, body mass index; CI, confidence interval; PTV, planning target volume; VIF, variance inflation factor.

Supplementary Table S2. ROC-based evaluation of optimal BED cutoffs (α/β ratios: 2–20) for predicting radiation-induced PTBE, with corresponding dose-per-fraction values for commonly applied fractionation schemes (1, 3, 5, 8, 10, 15, and 20 fractions) in all patients and age-stratified subgroups with convexity, parasagittal, and falcine meningiomas.

α/β	AUC	p-value	Optimal BED (Gy)	Sensitivity	Specificity	Youden's J	1 fx dose ≤ (Gy)	3 fx dose/fx ≤ (Gy)	5 fx dose/fx ≤ (Gy)	8 fx dose/fx ≤ (Gy)	10 fx dose/fx ≤ (Gy)	15 fx dose/fx ≤ (Gy)	20 fx dose/fx ≤ (Gy)
**All patients**													
2	0.550	0.551	119.840	0.500	0.804	0.304	14.51	7.99	5.99	4.56	4.00	3.12	2.60
3	0.556	0.503	89.427	0.500	0.804	0.304	14.95	8.07	5.98	4.48	3.89	2.99	2.46
4	0.559	0.476	74.550	0.500	0.804	0.304	15.38	8.17	5.98	4.42	3.82	2.89	2.35
5	0.593	0.267	65.096	0.500	0.784	0.284	15.71	8.21	5.95	4.35	3.73	2.79	2.25
6	0.602	0.219	59.672	0.500	0.804	0.304	16.16	8.33	5.98	4.33	3.69	2.73	2.19
7	0.662	0.051	50.457	0.688	0.667	0.355	15.62	7.90	5.60	4.01	3.40	2.48	1.97
8	0.663	0.050	48.025	0.688	0.667	0.355	16.00	8.00	5.64	4.00	3.38	2.45	1.93
9	0.675	0.036	46.798	0.688	0.686	0.374	16.51	8.17	5.72	4.04	3.40	2.45	1.93
10	0.726	0.007	43.785	0.812	0.667	0.479	16.51	8.07	5.61	3.93	3.29	2.36	1.85
11	0.730	0.006	42.577	0.812	0.667	0.479	16.83	8.15	5.63	3.92	3.28	2.34	1.83
12	0.733	0.005	41.075	0.812	0.667	0.479	17.00	8.15	5.60	3.88	3.24	2.30	1.79
13	0.718	0.009	41.217	0.750	0.725	0.475	17.54	8.36	5.72	3.95	3.29	2.33	1.81
**14**	**0.716**	**0.009**	**40.595**	**0.750**	**0.745**	**0.495**	**17.85**	**8.44**	**5.75**	**3.96**	**3.29**	**2.32**	**1.80**
**15**	**0.722**	**0.008**	**39.565**	**0.750**	**0.745**	**0.495**	**17.99**	**8.44**	**5.73**	**3.92**	**3.25**	**2.29**	**1.77**
16	0.719	0.009	38.136	0.750	0.725	0.475	17.96	8.35	5.64	3.84	3.18	2.23	1.72
17	0.680	0.031	38.487	0.625	0.804	0.429	18.45	8.54	5.75	3.91	3.23	2.26	1.75
18	0.677	0.034	37.910	0.625	0.804	0.429	18.63	8.56	5.75	3.90	3.22	2.25	1.73
19	0.682	0.029	37.394	0.625	0.804	0.429	18.80	8.59	5.74	3.88	3.20	2.23	1.71
20	0.654	0.065	36.749	0.625	0.804	0.429	18.90	8.57	5.72	3.85	3.17	2.21	1.69

**Patients younger than 70 years**													
α/β	AUC	p-value	Optimal BED (Gy)	Sensitivity	Specificity	Youden's J	1 fx dose ≤ (Gy)	3 fx dose/fx ≤ (Gy)	5 fx dose/fx ≤ (Gy)	8 fx dose/fx ≤ (Gy)	10 fx dose/fx ≤ (Gy)	15 fx dose/fx ≤ (Gy)	20 fx dose/fx ≤ (Gy)
2	0.745	0.082	144.118	0.800	0.871	0.671	16.01	8.85	6.66	5.09	4.46	3.50	2.93
3	0.745	0.082	105.562	0.800	0.871	0.671	16.36	8.88	6.60	4.97	4.32	3.33	2.75
4	0.758	0.067	95.031	0.800	0.903	0.703	17.60	9.43	6.95	5.18	4.48	3.42	2.80
5	0.790	0.040	80.775	0.800	0.903	0.703	17.75	9.37	6.83	5.03	4.33	3.26	2.64
6	0.790	0.040	70.271	0.800	0.903	0.703	17.75	9.23	6.66	4.86	4.15	3.09	2.48
7	0.906	0.004	50.457	1.000	0.710	0.710	15.62	7.90	5.60	4.01	3.40	2.48	1.97
8	0.906	0.004	48.025	1.000	0.710	0.710	16.00	8.00	5.64	4.00	3.38	2.45	1.93
9	0.919	0.003	46.798	1.000	0.742	0.742	16.51	8.17	5.72	4.04	3.40	2.45	1.93
10	0.919	0.003	45.160	1.000	0.742	0.742	16.83	8.25	5.74	4.02	3.38	2.42	1.90
11	0.939	0.002	44.440	1.000	0.839	0.839	17.28	8.40	5.81	4.06	3.40	2.43	1.90
**12**	**0.939**	**0.002**	**43.327**	**1.000**	**0.871**	**0.871**	**17.58**	**8.47**	**5.83**	**4.05**	**3.38**	**2.41**	**1.87**
**13**	**0.932**	**0.002**	**42.415**	**1.000**	**0.871**	**0.871**	**17.86**	**8.53**	**5.85**	**4.04**	**3.37**	**2.39**	**1.86**
**14**	**0.945**	**0.002**	**41.079**	**1.000**	**0.871**	**0.871**	**17.98**	**8.51**	**5.81**	**3.99**	**3.32**	**2.35**	**1.82**
15	0.932	0.002	39.565	1.000	0.839	0.839	17.99	8.44	5.73	3.92	3.25	2.29	1.77
16	0.919	0.003	38.136	1.000	0.806	0.806	17.96	8.35	5.64	3.84	3.18	2.23	1.72
17	0.719	0.120	39.565	0.600	0.935	0.535	18.79	8.72	5.88	4.00	3.31	2.32	1.79
18	0.706	0.143	38.700	0.600	0.935	0.535	18.89	8.70	5.84	3.96	3.27	2.29	1.76
19	0.706	0.143	37.926	0.600	0.935	0.535	18.98	8.68	5.81	3.93	3.24	2.26	1.74
20	0.623	0.385	36.749	0.600	0.903	0.503	18.90	8.57	5.72	3.85	3.17	2.21	1.69
**Patients older than 70 years**													
α/β	AUC	p-value	Optimal BED (Gy)	Sensitivity	Specificity	Youden's J	1 fx dose ≤ (Gy)	3 fx dose/fx ≤ (Gy)	5 fx dose/fx ≤ (Gy)	8 fx dose/fx ≤ (Gy)	10 fx dose/fx ≤ (Gy)	15 fx dose/fx ≤ (Gy)	20 fx dose/fx ≤ (Gy)

2	0.468	0.773	98.075	0.273	0.950	0.223	13.04	7.15	5.34	4.05	3.54	2.75	2.29
3	0.473	0.804	72.450	0.182	1.000	0.182	13.32	7.14	5.26	3.92	3.40	2.59	2.12
4	0.473	0.804	61.838	0.182	1.000	0.182	13.85	7.30	5.31	3.91	3.36	2.53	2.05
5	0.477	0.836	55.470	0.182	1.000	0.182	14.34	7.43	5.36	3.90	3.33	2.47	1.99
6	0.486	0.901	54.792	0.545	0.600	0.145	15.38	7.89	5.65	4.08	3.47	2.56	2.04
7	0.536	0.741	51.450	0.545	0.600	0.145	15.80	8.00	5.68	4.07	3.45	2.52	2.00
8	0.536	0.741	50.169	0.455	0.700	0.155	16.43	8.24	5.81	4.13	3.49	2.54	2.01
9	0.550	0.650	44.939	0.909	0.300	0.209	16.11	7.95	5.56	3.91	3.29	2.37	1.86
10	0.600	0.364	43.785	0.727	0.600	0.327	16.51	8.07	5.61	3.93	3.29	2.36	1.85
11	0.586	0.433	42.577	0.727	0.600	0.327	16.83	8.15	5.63	3.92	3.28	2.34	1.83
12	0.595	0.386	41.075	0.727	0.600	0.327	17.00	8.15	5.60	3.88	3.24	2.30	1.79
13	0.577	0.483	40.719	0.636	0.600	0.236	17.41	8.29	5.67	3.91	3.26	2.31	1.79
14	0.564	0.563	39.989	0.636	0.600	0.236	17.67	8.35	5.69	3.91	3.25	2.29	1.77
15	0.582	0.457	40.237	0.545	0.750	0.295	18.19	8.54	5.80	3.98	3.30	2.32	1.80
16	0.582	0.457	39.659	0.545	0.750	0.295	18.43	8.60	5.82	3.97	3.29	2.31	1.78
17	0.591	0.409	39.150	0.545	0.750	0.295	18.66	8.65	5.83	3.97	3.28	2.30	1.77
18	0.591	0.409	38.617	0.545	0.750	0.295	18.86	8.68	5.83	3.96	3.27	2.28	1.76
19	0.591	0.409	38.084	0.545	0.750	0.295	19.03	8.71	5.83	3.94	3.25	2.27	1.74
20	0.595	0.386	37.605	0.545	0.750	0.295	19.19	8.73	5.82	3.93	3.24	2.25	1.73

AUC, area under the curve; BED, biologically effective dose; Fx, fraction; Gy, gray; PTBE, peritumoral brain edema; ROC, receiver operating characteristic curve; Youden's J, Youden index (J = sensitivity + specificity – 1).

Supplementary Table S3. Comparison between patients with and without the development of PTBE after radiotherapy among those with pre-existing edema prior to radiotherapy.

Characteristics	PTBE (-)	PTBE (+)	Total	p-value
Number (%)	4 (50.0)	4 (50.0)	8	
Sex, female, n (%)	2 (50.0)	1 (25.0)	3 (37.5)	0.465
Age, mean ± SD, y	76.3 ± 6.9	82.5 ± 5.7	79.4 ± 6.8	0.215
GTV, mean ± SD, y	22.8 ± 20.3	39.2 ± 9.6	31.0 ± 17.1	0.194
PTV, mean ± SD, y	25.9 ± 28.4	49.1 ± 15.5	37.5 ± 24.5	0.203
BED ($\alpha/\beta = 3$), mean ± SD, Gy	80.6 ± 4.2	76.1 ± 9.8	78.4 ± 7.4	0.428

BED, biologically effective dose; GTV, gross tumor volume; PTBE, peritumoral brain edema; PTV, planning target volume; SD, standard deviation.
